# Enhanced antibody-mediated neutralization of HIV-1 variants that are resistant to fusion inhibitors

**DOI:** 10.1186/s12977-016-0304-7

**Published:** 2016-09-27

**Authors:** Muntasir Alam, Takeo Kuwata, Kazuya Shimura, Masaru Yokoyama, Kristel Paola Ramirez Valdez, Kazuki Tanaka, Yasuhiro Maruta, Shinya Oishi, Nobutaka Fujii, Hironori Sato, Masao Matsuoka, Shuzo Matsushita

**Affiliations:** 1 Matsushita Project Laboratory, Center for AIDS Research, Kumamoto University, 2-2-1 Honjo, Chuo-ku, Kumamoto, 860-0811 Japan; 2 Laboratory of Virus Control, Institute for Virus Research, Kyoto University, Kyoto, Japan; 3 Pathogen Genomics Center, National Institute of Infectious Diseases, Tokyo, Japan; 4 Graduate School of Pharmaceutical Sciences, Kyoto University, Kyoto, Japan

**Keywords:** HIV, Fusion inhibitor resistance, Neutralization sensitivity

## Abstract

**Background:**

HIV-1 typically develops resistance to any single antiretroviral agent. Combined anti-retroviral therapy to reduce drug-resistance development is necessary to control HIV-1 infection. Here, to assess the utility of a combination of antibody and fusion inhibitor treatments, we investigated the potency of monoclonal antibodies at neutralizing HIV-1 variants that are resistant to fusion inhibitors.

**Results:**

Mutations that confer resistance to four fusion inhibitors, enfuvirtide, C34, SC34, and SC34EK, were introduced into the envelope of HIV-1_JR-FL_, a CCR5-tropic tier 2 strain. Pseudoviruses with these mutations were prepared and used for the assessment of neutralization sensitivity to an array of antibodies. The resulting neutralization data indicate that the potencies of some antibodies, especially of those against the CD4 binding site, V3 loop, and membrane-proximal external region epitopes, were increased by the mutations in gp41 that conferred resistance to the fusion inhibitors. C34-, SC34-, and SC34EK-resistant mutants showed more sensitivity to monoclonal antibodies than enfuvirtide-resistant mutants. An analysis of C34-resistant mutations revealed that the I37K mutation in gp41 HR1 is a key mutation for C34 resistance, low infectivity, neutralization sensitivity, epitope exposure, and slow fusion kinetics. The N126K mutation in the gp41 HR2 domain contributed to C34 resistance and neutralization sensitivity to anti-CD4 binding site antibodies. In the absence of L204I, the effect of N126K was antagonistic to that of I37K. The results of a molecular dynamic simulation of the envelope trimer confirmation suggest that an I37K mutation induces the augmentation of structural fluctuations prominently in the interface between gp41 and gp120. Our observations indicate that the “conformational unmasking” of envelope glycoprotein by an I37K mutation is one of the mechanisms of neutralization sensitivity enhancement. Furthermore, the enhanced neutralization of C34-resistant mutants in vivo was shown by its high rate of neutralization by IgG from HIV patient samples.

**Conclusions:**

Mutations in gp41 that confer fusion inhibitor resistance exert enhanced sensitivity to broad neutralizing antibodies (e.g., VRC01 and 10E8) and other conventional antibodies developed in HIV-1 infected patients. Therefore, next-generation fusion inhibitors and monoclonal antibodies could be a potential combination for future regimens of combined antiretroviral therapy.

**Electronic supplementary material:**

The online version of this article (doi:10.1186/s12977-016-0304-7) contains supplementary material, which is available to authorized users.

## Background

Combined anti-retroviral therapy (ART) has been effective at suppressing HIV-1 replication, but ART is unable to cure HIV-1 infection and demands a lifetime investment [1, 2]. Additionally, the long-term application of ART is associated with several adverse effects and the emergence of drug resistance [3–5]. To avoid these issues, the development of a new ART combination that targets different steps of viral replication other than reverse transcription, integration, and protease processing of viral protein should be pursued. For example, HIV-1 entry inhibitors could be an attractive target for investigation.

Stepwise knowledge regarding viral entry, which involves attachment with CD4, binding with a co-receptor (CCR5 or CXCR4), and fusion with the target cell, has allowed to develop diverse antiviral agents, such as *N*-phenyl-*N*-piperidin-4-yl-oxalamide, BMS-663068, AMD3100, Maraviroc, and Cenicriviroc [6–8]. Among entry inhibitors, the structural features of HIV-1 fusion machinery in gp41 have helped to develop fusion inhibitors. During HIV-1 entry, after co-receptor binding, conformational changes trigger the formation of a six-helix bundle composed of an N-terminal heptad repeat (HR1) and a C-terminal heptad repeat (HR2). Fusion inhibitors are peptides corresponding to the HR1 or HR2 region that bind with another counterpart and inhibit the formation of the six-helix complex. The classic fusion inhibitors are HR2-derived peptides, such as enfuvirtide (ENF) and C34, among which only ENF has received FDA approval for clinical application [6]. ENF has less toxicity, and its co-administration with tipranavir, darunavir, and maraviroc significantly improved response rates [9]. The major obstacle for the therapeutic application of ENF is the rapid development of resistance, especially when it is delivered without a strong regimen. Notably, a single point mutation can emerge within several weeks after ENF administration that can give rise to drug resistance [10–13]. In contrast, C34 imposes a relatively high genetic barrier for resistance development in vitro [14]. Derivatives of C34 peptides have been developed with better solubility, enhanced α-helicity, enhanced activity, and a bigger barrier to resistance development [15]. These C34 derivatives include SC34 and SC34EK, which are still under pre-clinical evaluation [16, 17].

Within a few weeks of detectable HIV-1 viremia, an antibody response develops in infected persons [18]. The antibodies developed against HIV-1 envelope have the potential to target all steps of viral entry. A range of recently discovered broad neutralizing antibodies (bnAbs) indicates the possibility of antibody-mediated immunotherapy, which could inhibit diverse HIV-1 subtypes. Several of these monoclonal antibodies (MAbs) were found to control viral infection in humanized mice and in macaques [19, 20]. Recently, clinical trials in HIV-1-infected patients have shown encouraging effects for antibody-mediated suppression of viral replication in vivo. Antibodies such as 3BNC117, VRC01, and KD247 were well tolerated in human trials and each maintained a 1–2.5-log reduction of viral load for 30–100 days [21–24]. However, like all other anti-retroviral drugs, the single administration of an antibody is prone to resistance development. Hence, combination strategies are necessary.

Here, we report enhanced neutralization sensitivity to an array of neutralizing antibodies, including bnAbs, due to mutations in gp41 that confer C34, SC34, or SC34EK resistance. A decade ago, Reeves et al. reported changes in the neutralization sensitivity of the ENF-resistant mutants against some neutralizing antibodies [25]. In the present study, we report that recently developed bnAbs, such as VRC01 and 10E8, can neutralize fusion inhibitor-resistant mutants efficiently. Furthermore, conventional antibodies against the CD4 binding site (CD4bs) and V3 loop become potent against drug-resistant mutants of HIV-1 compared with their effects against wild-type (WT) virus. Along with neutralization sensitivity, we have identified a mutation (I37K) in the gp120–gp41 interactive region of gp41 HR1 that has a global impact on the different antigenic sites on the HIV-1 envelope. The results will add new insights to our understanding of the HIV-1 envelope and may help to select antibody partners for combined therapy with fusion inhibitors.

## Methods

### Patient samples

Plasma samples from HIV-1-infected patients, including patient KTS376 described in our previous study, were collected and purified using protein A-Sepharose (Affi-gel Protein A; Bio-Rad, Hercules, CA, USA) [26].

### Cells, plasmids, and antibodies

TZM-bl [27] and 293T [28] cells were maintained in Dulbecco’s modified Eagle medium (DMEM; Sigma, St. Louis, MO, USA) supplemented with 10 % heat-inactivated fetal calf serum (FCS; Thermo Scientific, Waltham, MA). The generation of antibodies 49G2, 82D5, 42F9, 0.5γ, KD-247, 16G6, 916B2, and 4E9C were previously reported by Ramirez Valdez et al. [26]. The antibody 2E8S34 was similarly isolated by EBV transformation from patient KTS376 [26]. Plasmids for the expression of heavy and light chains of bnAb VRC01 were obtained through the NIH AIDS Reagent Program, Division of AIDS, NIAID, NIH from Dr. J. Mascola [22]. bnAb b12 [29] was kindly provided from Dr. D. Burton. Heavy and light chain genes of 10E8 [30] and 2G12 [31] were synthesized from their amino acid sequences (GeneArt Strings DNA Fragments; Invitrogen, Carlsbad, CA, USA) and inserted into expression vectors as described previously [26]. Soluble CD4 was purchased commercially (sCD4, R&D systems, Inc., Minneapolis, MN, USA). Anti-membrane-proximal external region (MPER) antibodies 4E10 [32] and 2F5 [33] were purchased commercially (Polymun Scientific, GmbH, Klosterneuburg, Austria). Fusion inhibitors, C34, SC34, SC34EK, and ENF were synthesized following the methods described in a previous report [16].

### Construction of recombinant plasmids and mutants

Plasmids to express mutant envelopes, pCXN-JR-FL_V38A_, pCXN-JR-FL_Q40H_, pCXN-JR-FL_N43D_, pCXN-JR-FL_C34r_ (I37K/N126K/L204I), pCXN-JR-FL_SC34r_ (I37K/R46K/Q52R/Q56R/N126K/S138A/E151K/K154N/L204I/L210F), and pCXN-JR-FL_SC34EKr_ (Q41R/N43K/A96D/N126K/V182I/P203S/L204I/H258Q/A312T) were constructed by oligonucleotide-based site-directed mutagenesis with pCXN-JR-FL (kindly provided by Dr. Y. Maeda, Kumamoto University), which express WT JR-FL envelope [15]. Single and double mutants of C34r mutations, I37K, N126K, and L204I, were constructed by PCR and subsequent homologous recombination using the GeneArt^®^ seamless cloning an assembly enzyme mix (Invitrogen).

The plasmid expressing both the JR-FL envelope and IRES2-EGFP, pCXN-JR-FL-IRES2-EGFP was constructed by inserting the IRES2-EGFP fragment, which was amplified by primers 5IRES2 (5′-GGTGGGAGCAGTATCTCGAGGATCCGCCCCTC-3′) and 3EGFP2 (5′-CGGCTTTTCCAGGTCTTTACTTGTACAGCTCG-3′) using pLp-IRES2-EGFP [34] as the template, into the XhoI site of pCXN-JR-FL. Plasmids to express the other envelope mutants and EGFP (enhanced green fluorescent protein) were similarly constructed and used for flow cytometry studies.

### Pseudovirus preparation

Pseudoviruses were prepared as described previously [26]. Briefly, exponentially dividing 293T cells were transfected with 1.3 µg of pSG3^ΔEnv^ and 5.2 µg of envelope expression vector using Lipofectamine 2000 transfection reagent (Invitrogen, Life Technologies, Carlsbad, CA, USA) in six-well cell culture plates. After 48 h of transfection, pseudovirus-containing supernatants were harvested, filtered through a 0.2-µm pore-size filter, and stored at −80 °C until further use. The median tissue culture infectious dose (TCID_50_) of each pseudovirus was determined using TZM-bl cells. The amount of p24 was determined by using a commercial enzyme-linked immunosorbent assay (ELISA) kit (ZeptoMetrix Corporation, Buffalo, NY) according to the manufacturer’s instructions.

### Analysis of sensitivity to antibodies, sCD4, and fusion inhibitors

The neutralization sensitivities of pseudoviruses were measured following a previously described protocol [35]. Briefly, serial dilutions of MAbs, sCD4, or fusion inhibitors in DMEM with 10 % FCS were placed in 96-well cell culture plates in triplicate. After dilution, 200 TCID_50_ of each respective pseudovirus suspension was added to each well. Each plate had triplicates of the respective viral control (without any inhibitor) and a cell control (without any virus). The virus-MAb mixture was incubated for 1 h (37 °C, 5 % CO_2_). After incubation, 10^4^ TZM-bl cells in suspension with 30 µg/mL of DEAE-dextran were added to each well and incubated for 48 h (37 °C, 5 % CO_2_). Finally, the cells were washed, lysed, and the firefly luciferase activity was measured using a Galacto-star system (Life Technologies, Carlsbad, CA, USA). The percent inhibition by MAb, sCD4, or fusion inhibitor was determined by comparing the RLU in the presence and absence of an inhibitor. Each assay was repeated at least three times and validated according to the pass/fail criteria for TZM-bl cell based neutralization assay described by Sarzotti-Kelsoe et al. [36].

### Antibody-envelope binding assays

The binding of MAbs to the HIV-1 envelope that was expressed on transiently transfected 293T cells was analyzed by flow cytometry. One day before transfection, 293T cells were cultured (2 × 10^6^ cells/well) in six-well plates. When the cell growth reached about 60–70 % confluency, 1 µg of plasmid expressing both the envelope and EGFP was transfected using Lipofectamine 2000 transfection reagent (Invitrogen). After 48 h of transfection, the cells were washed with phosphate-buffered saline (PBS) and harvested with 0.05 % trypsin and resuspended in PBS containing 0.2 % BSA. For each envelope mutant, 10^5^ cells were stained with the respective primary antibody for 30 min at room temperature (RT). After incubation, the cells were washed twice with PBS containing 0.2 % BSA and incubated with allophycocyanin-conjugated AffiniPure F(ab’)_2_ Fragment Goat Anti-Human IgG (H + L) (Jackson ImmunoResearch, West Grove, PA, USA) for 15 min at RT. Cells were fixed with PBS containing 10 % formalin and analyzed using the FACSCalibur system (Becton–Dickinson, Franklin Lakes, NJ, USA). After gating on the EGFP^+^ cells, the mean fluorescence intensity (MFI) for each sample was calculated using FlowJo (TreeStar, San Carlos, CA, USA). Each mutant was stained with 2G12 (which targets the glycan structure on HIV-1 envelope) for normalization, and the antibody–envelope binding was represented by the antibody MFI/2G12 MFI ratio [37].

### Comparison of envelope content on virion surfaces

To compare the envelope content per virion, we determined the amount of p24 and gp120 in a pseudovirus stock by ELISA. As negative control, delta envelope pseudovirus (pSG3^ΔEnv^ only) was used in the experiment. Pseudovirus stocks were centrifuged at 13,200 rpm for 90 min at 4 °C, and the viral pellets were resuspended in 1 mL of PBS and centrifuged again under the same conditions. Finally, the viral pellets were lysed with Tris-buffered saline containing 1 % empigen (Sigma). The amount of p24 protein was determined with an ELISA kit (ZeptoMetrix Corporation, Buffalo, NY, USA) as described above. For determination of the amount of gp120, ELISA plates were coated with 10 µg/ml of anti-V3 specific antibody 0.5γ in carbonate-bicarbonate buffer overnight at 4 °C. The plate was washed with ELISA wash buffer (PBS with 0.02 % Tween20) and blocked at RT for 2 h with PBS containing 3 % BSA. After blocking, the plates were washed twice, 50 µL of viral lysate was added to each specific well, and they were incubated at RT for 3 h. The gp120 plates were subsequently washed three times and incubated with 2G12 (1.5 µg/mL) at RT for 2 h. Next, the plate was washed three times with ELISA wash buffer, and alkaline phosphatase-conjugated anti-human IgG (1:1000, Sigma) was added to each well and incubated for another 2 h. Finally, phosphatase substrate (Sigma) was added, and the absorbance was determined using an ELISA reader (Model 680 microplate reader, Bio-Rad) at 405 nm. A standard curve was plotted using a serial dilution of purified HIV-1 SF2 gp120. The envelope content per virion was represented by the ratio of gp120/p24 concentration [38].

### Envelope fusion kinetics

The fusion kinetics of WT and mutant envelope proteins were determined using dual split protein (DSP)-dependent cell–cell fusion assays with a protocol adapted from Kondo et al. [39]. Plasmids for this assay, pDSP_1–7_ and pDSP_8–11_, were kindly provided by Dr. Z. Matsuda, The University of Tokyo. Briefly, for the generation of HIV-1 envelope-expressing cells, 293T cells in a six-well cell culture plate were co-transfected with pDSP_1–7_ and a plasmid expressing both envelope (Env) and EGFP. Concurrently, TZM-bl cells (10^4^ cells/well) were transfected with pDSP_8–11_ plasmid in a 96-well white-bottomed cell culture plate. After 36 h of transfection, the TZM-bl cell media were replaced with 50 µL of fresh DMEM with 10 % FCS containing 60 µM of EnduRen (Promega Corporation, Madison, WI, USA), and the cells were incubated 2 h at 37 °C in 5 % CO_2_. At the end of the incubation, 293T cells were harvested using 0.05 % trypsin and resuspended at a concentration of 2 × 10^6^ cells/mL in fresh medium. For co-culture, 50 µL of resuspended Env-DSP_1–7_-expressing 293T cells were transferred into the wells of CD4-CCR5-DSP_8–11_-expressing TZM-bl cells and mixed very gently several times. *Renilla* luciferase activity was measured with a luminometer at 0, 15, 30, 45, 60, 75, 90, and 120 min time-points after co-culture. During co-culture, the expression level of envelope on the transfected cells was analyzed by staining with 2G12. The expression levels of envelope mutants were confirmed to be similar to that of WT envelope (<20 % change in MFI). The fusion percentage was calculated using the RLU value at 120 min as 100 %.

### Molecular dynamic (MD) simulations of the HIV-1 gp41 trimer

The extracellular portion of the HIV-1_JR-FL_ gp41 structures with and without an I37K mutation were constructed by using the homology modeling method with Molecular Operating Environment (Chemical Computing Group Inc., Montreal, QC, Canada). The crystal structure of the HIV-1 BG505 SOSIP.664 gp140 trimer at a resolution of 3.1 Å (PDB code: 4TVP) [40], which contains the extracellular portion of the gp41 trimer in association with the gp120 trimer, was used as the modeling template. MD simulations were performed as previously described to analyze changes in the structural dynamics of protein interaction of the surface in solution [41–45]. The simulations were done by the pmemd module in the Amber 11 program package [46] with the AMBER ff99SB-ILDN force field [47] and the TIP3P water model for simulations of aqueous solutions [48]. A non-bonded cutoff of 10 Å was used. Bond lengths involving hydrogen were constrained with SHAKE, a constraint algorithm to satisfy Newtonian motion [49], and the time step for all MD simulations was set to 2 fs. After heating calculations for 20 ps until 310K using the NVT ensemble, simulations were executed using the NPT ensemble at 1 atm, at 310K, and in 150 mM NaCl for 100 ns. Root mean square fluctuation (RMSF) were calculated as previously described [41–45] to quantify the structural dynamics of the molecules in these MD simulations. RMSF of the Cα atoms were calculated to obtain information about the atomic fluctuations of individual amino acid residues during MD simulations [46]. The 2000 snapshots obtained from MD simulations of 80–100 ns were used to calculate RMSF. The average structures were used as reference structures for RMSF calculation. RMSF, which quantifies the differences between the average values and those obtained at given times of MD simulations, was calculated using the ptraj module in Amber, a trajectory analysis tool [46].

## Results

### Enhanced neutralization of C34-, SC34-, and SC34EK-resistant mutants compared with WT and ENF-resistant mutants

We selected HIV-1 strain JR-FL, which is a primary CCR5-tropic isolate that has been classified in the tier 2 level of neutralization sensitivity, to use as our WT for evaluating the neutralization sensitivity of drug-resistant mutants. The Env of JR-FL is relevant to subtype B clinical isolates and has been used as the WT strain for mutagenesis analyses in many previous studies [50–52]. Mutants resistant to C34, SC34, and SC34EK were previously obtained by in vitro passages of the NL4-3 strain in the presence of each inhibitor (Additional file 1: Fig. S1) [53–55]. For comparison with other fusion inhibitor resistant mutants, we selected three ENF resistant mutants V38A, Q40H, and N43D, because these mutations were frequently observed in ENF treated patients and confer more than tenfold resistance to ENF [54, 56, 57]. We constructed fusion inhibitor-resistant mutants by inserting each mutation associated with ENF, C34, SC34, or SC34EK resistance into the Env construct with a JR-FL background (pCXN-JR-FL), and the ENF-resistant clones were designated as V38A, Q40H, and N43D, the C34-resistant clone was designated as C34r, the SC34-resistant clone was designated as SC34r, and the SC34EK-resistant clone was designated as SC34EKr. We prepared pseudoviruses with Env from JR-FL WT or from variants with mutations conferring fusion inhibitor resistance and tested their sensitivity to the corresponding fusion inhibitors (Additional file 2: Fig. S2). We confirmed the complete resistance to the corresponding inhibitors of JR-FL-based pseudoviruses with mutations conferring resistance to ENF, C34, SC34, or SC34EK. The resulting data show the successful transfer of the resistance phenotype to a JR-FL background. Concerning C34 resistance, we also constructed additional three mutants, I37K, N126K, and N126K/L204I, and we found that I37K contributed most of the resistance to C34 (Additional file 2: Fig. S2).

**Fig. 1:**
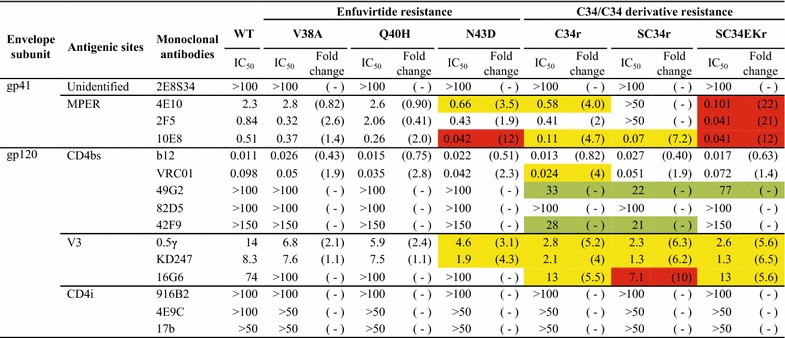
Neutralization sensitivities of mutants resistant to ENF, C34, SC34, or SC34EK. The neutralization sensitivities of fusion inhibitor-resistant mutants are shown as the average IC_50_ (µg/mL) of MAbs from several independent experiments. The fold change in neutralization sensitivity with respect to WT is presented in *brackets* below each IC_50_ values and highlighted by the following *color code*: *yellow*; 3–10-fold, *red*; >10 fold, and *green*; an emergence of neutralization sensitivity for MAbs that are not neutralizing to WT virus. Mutants for which calculation of fold change was not possible are presented with “–” in *brackets*

To investigate the effect on antibody-mediated HIV-1 neutralization of mutations that confer fusion inhibitor resistance, we examined the sensitivity of fusion inhibitor-resistant pseudoviruses to a panel of MAbs targeting CD4bs, V3 loop, CD4i (CD4 induced epitope), or MPER epitopes. We observed enhanced neutralization sensitivity of C34-, SC34-, and SC34EK-resistant viruses by the selected antibodies with respect to the neutralization sensitivity of the WT virus (Fig. 1). In contrast, two of the three mutations conferring ENF resistance, V38A and Q40H, were unable to affect the neutralization sensitivity by these antibodies. The N43D mutation conferring ENF resistance enhanced the neutralization by anti-MPER antibodies 4E10 (>threefold) and 10E8 (>tenfold). These data are comparable to those of a previous report that showed HR1 mutations conferring ENF resistance enhance the neutralization by anti-MPER antibodies 4E10 and 2F5 [25]. In addition to the enhanced neutralization sensitivity to anti-MPER antibodies of the N43D mutant, we also observed an over threefold enhancement of neutralization by anti-V3 antibodies 0.5γ and KD247 for this mutant (Fig. 1).

The C34r with I37K/N126K/L204I mutations was sensitive to neutralization by 4E10 and 10E8 (>threefold). Both anti-CD4bs bnAb b12 and VRC01, potently neutralized both WT and C34r mutants. Interestingly, 49G2 and 42F9, which are non-neutralizing antibodies against the WT virus, were able to neutralize the C34r mutant. Furthermore, the C34r mutant was more sensitive to all anti-V3 antibodies than the WT virus.

The SC34r mutant was not sensitive to neutralization by the anti-MPER antibodies 4E10 and 2F5 but was neutralized by 10E8 with a higher potency (>threefold) than the WT virus. The presence of E151K and K154N mutations in the 2F5 epitope ELDKWA may explain why SC34r is not sensitive to 2F5 (Additional file 1: Fig. S1). Enhanced neutralization by anti-CD4bs and anti-V3 antibodies was observed in the SC34r mutant, similar to the enhanced neutralization of these antibodies in the C34r mutant. The SC34EKr variant had the highest enhancement of neutralization sensitivity for all anti-MPER antibodies as compared with other fusion inhibitor-resistant mutants. Although the anti-CD4bs antibody 42F9 was unable to neutralize the SC34EKr mutant, neutralization enhancement in this mutant was observed for 49G2 and all three anti-V3 antibodies (Fig. 1). For CD4i MAbs, subtle differences in the neutralization sensitivities were observed between the WT and mutants. In general, our observations indicate that mutations conferring fusion inhibitor resistance also confer enhanced neutralization sensitivity to the CD4bs, V3 loop, and MPER epitopes.

### C34-resistance-conferring mutations I37K and N126K are critical for increasing the sensitivity to antibodies directed against either gp120 or gp41

**Fig. 2:**
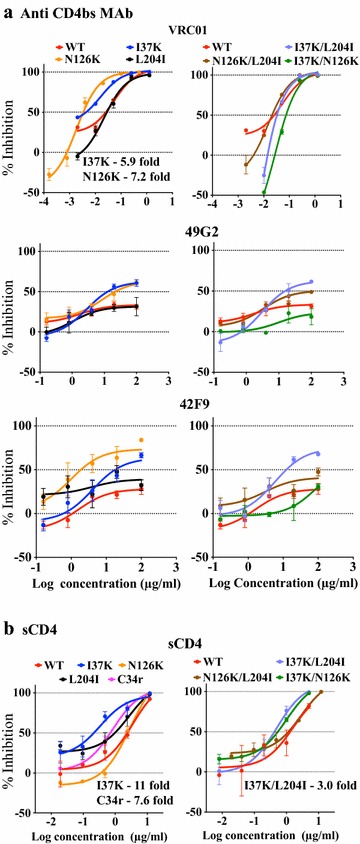
Effect of C34 resistance-conferring mutations on the sensitivities to anti-CD4bs MAb and sCD4. Three mutations that together confer C34 resistance, I37K, N126K, and L204I, were examined for their effect on the neutralization sensitivity to anti-CD4bs MAbs, VRC01, 49G2, and 42F9, (**a**) and to sCD4 (**b**) using single (*left*) and double (*right*) mutants. In each graph, the *x axis* represents the log concentration of MAbs in µg/mL and the *y axis* represents the percent inhibition compared with the corresponding no inhibitor control. The results are shown as the means ± SEs of three independent experiments

To investigate the effect on the neutralization sensitivity of mutations conferring fusion inhibitor resistance, we focused on the C34r mutant that comprises three mutations (I37K/N126K/L204I). The impact of the individual mutations in the C34r mutant on its sensitivity to antibodies was analyzed by performing neutralization assays using antibodies targeting the CD4bs (VRC01, 49G2, and 42F9), V3 (16G6, KD247, and 0.5γ), and MPER (10E8). As shown in Fig. 2a, I37K (5.9-fold) and N126K (7.2-fold) mutants are more sensitive to VRC01 than WT, based on their corresponding IC_50_ values. However, the neutralization sensitivity of the combination of these two mutations (I37K/N126K) was unaltered with respect to WT. Additionally, neutralization by VRC01 was unaffected by the combinations of mutations I37K/L204I and N126K/L204I compared to WT. Anti-CD4bs antibodies, 49G2 and 42F9, failed to neutralize WT virus, but the inhibition by these antibodies reached over 50 % against I37K, N126K, and I37K/L204I mutants. These results indicate that I37K and N126K are the key mutations responsible for increased neutralization sensitivity against anti-CD4bs antibodies, and that the combination of these mutations with L204I caused the C34-resistant phenotype in C34r. Interestingly, the I37K and N126K mutations were unable to increase the sensitivity to all of these anti-CD4bs antibodies in combination, suggesting that the effects of these mutations on the sensitivity of the viruses to anti-CD4bs antibodies were antagonized by each other (Fig. 2a).

To investigate the impact of sCD4 on both single and double mutants of C34-resistant variants, neutralization assays were performed with sCD4. The resulting data show that sensitivity to sCD4 was significantly higher in I37K (11-fold) and C34r (7.6-fold) mutants than in the WT virus. Furthermore, a slight increase in sCD4 sensitivity by I37K/L204I (threefold) was observed (Fig. 2b). This marked increase in the sensitivity to sCD4 in the I37K mutant indicates that the I37K mutation is predominantly responsible for the observed increase in sCD4 sensitivity of C34-resistant variants.

**Fig. 3:**
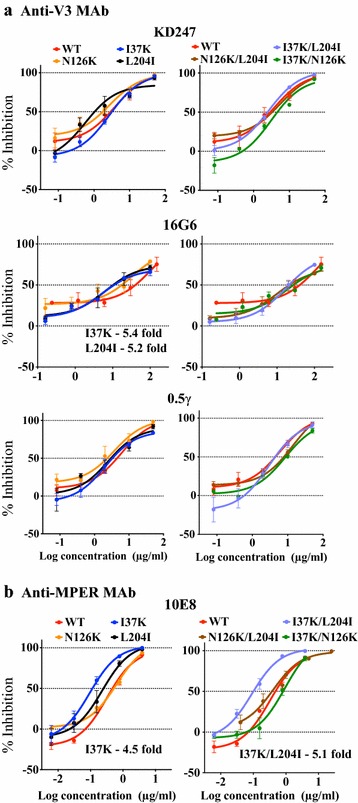
Effect of C34 resistance-conferring mutations on the sensitivities to MAbs against V3 and MPER. Three mutations that together confer C34 resistance, I37K, N126K, and L204I, were examined for their effect on the neutralization sensitivity to the anti-V3 MAbs, KD247, 16G6, and 0.5γ, (**a**) and to the anti-MPER MAb 10E8 (**b**) using single (*left*) and double (*right*) mutants. In each graph, the *x axis* represents the log concentration of MAbs in µg/ml and the *y axis* represents the percent inhibition compared with the corresponding no inhibitor control. The results are shown as the means ± SEs of three independent experiments

Although C34r was more sensitive to anti-V3 antibodies than WT (Fig. 1), single and double mutations of C34r did not affect its sensitivity to any of the tested anti-V3 antibodies except 16G6 (Fig. 3a). Mutants with I37K and L204I showed a fivefold increase in their sensitivity to 16G6; however, the sensitivities to KD247 and 0.5γ were similar between WT and mutants. This suggests that the combination of three mutations in C34r may act synchronously to impact the neutralization profile of anti-V3 antibodies (Fig. 3a).

The neutralization potency of the anti-MPER antibody 10E8 was enhanced in I37K (4.5-fold) and I37K/L204I (fivefold) mutants compared with WT (Fig. 3b). This neutralization pattern of I37K and I37K/L204I was similar to that of anti-CD4bs antibodies, 49G2 and 42F9 (Fig. 2a). These results demonstrate that the C34r mutation I37K plays a crucial role in the increased sensitivity to antibodies directed against CD4bs and MPER, even though the enhanced neutralization by anti-V3 antibodies requires all three C34-resistance-associated mutations. In contrast, the N126K mutation only influences the neutralization by anti-CD4bs antibodies.

### The I37K mutation enhances the binding affinity of antibodies to epitopes on gp120

**Fig. 4:**
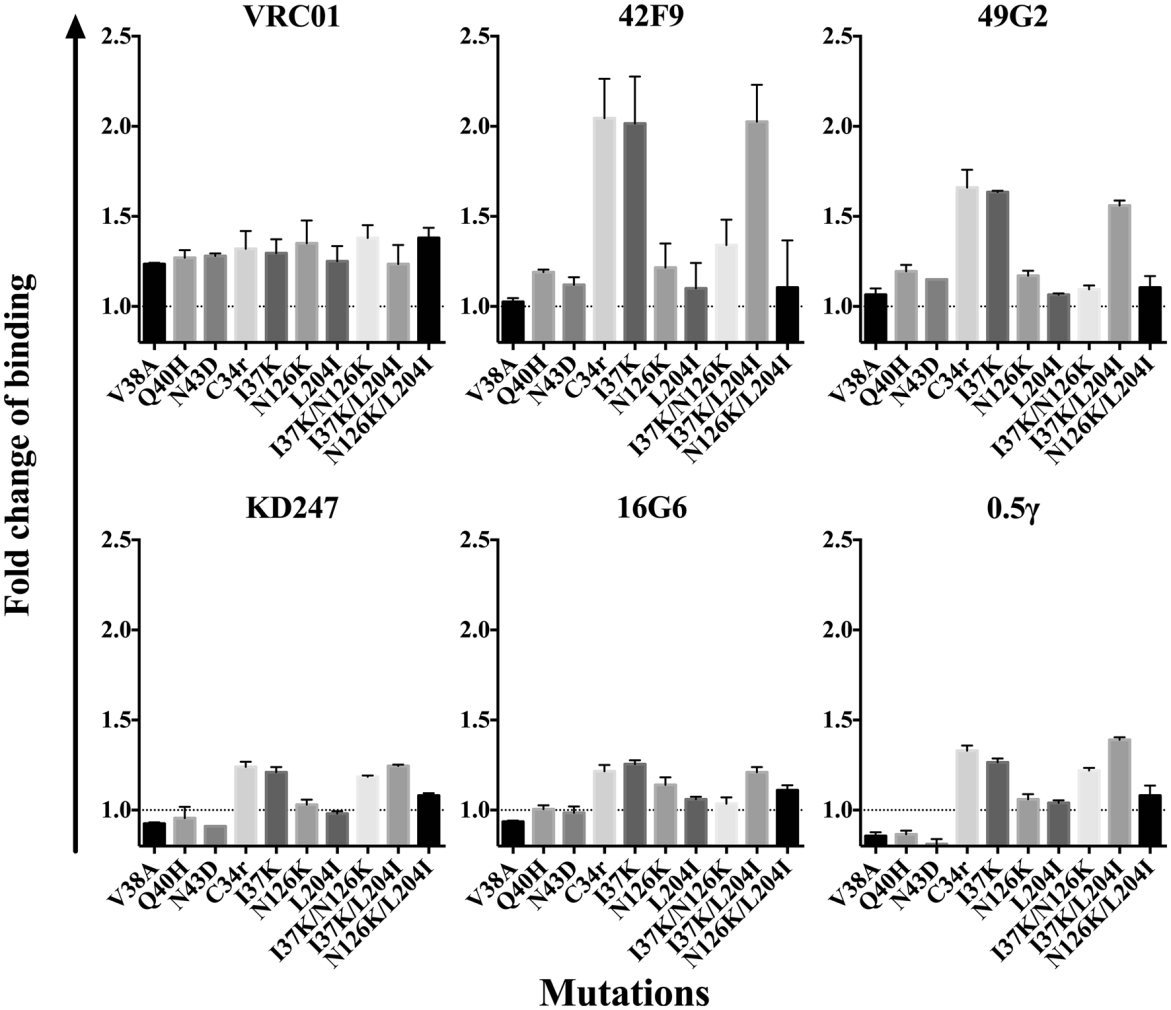
Binding of antibodies to untriggered envelope trimers. The effect of fusion inhibitor resistance-conferring mutations on the binding of anti-CD4bs MAbs, VRC01, 49G2, and 42F9, and of anti-V3 MAbs, KD247, 16G6, and 0.5γ, was determined by performing flow cytometry on 293T cells expressing mutant envelopes. The geometric MFI was calculated and normalized by the MFI of 2G12. The changes in antibody binding levels are presented as the fold change of binding with respect to WT. The *dash line* represents the change in antibody binding (fold change = 1). The results are shown as the means ± SEs of three replicas

To investigate the mechanism of neutralization enhancement, we used flow cytometry to assess the binding of antibodies against CD4bs (VRC01, 42F9, and 49G2) and V3 (KD247, 16G6, and 0.5γ) to mutant envelopes expressed on the cell surface and evaluated the exposure level of epitopes on un-triggered Env trimers (Fig. 4). A striking enhancement was observed for anti-CD4bs antibodies, 42F9 (twofold) and 49G2 (1.6–1.7 fold), against C34r, I37K and I37K/L204I mutants. These mutants all had the I37K mutation, suggesting that the increased binding affinity of 42F9 and 49G2 to viruses with I37K enhances the neutralization by these antibodies (Fig. 2a). N126K, the other C34-resistant mutation that is critical for enhanced neutralization, did not affect the antibody binding, and, instead, it abrogated the enhancement effect of I37K in the I37K/N126K mutant. This finding suggests that the N126K mutation enhances neutralization by a mechanism other than an increase in the affinity between the envelope and the antibody. We observed some increase in VRC01 binding, but this occurred in the absence of any correlation with neutralizing sensitivity (Fig. 4). This lack of correlation between neutralization sensitivity and binding affinity indicates that the enhanced neutralization sensitivity of I37K and N126K to VRC01 is mediated by some other unknown mechanism (Fig. 2a).

The I37K mutation also slightly affected the binding of anti-V3 antibodies, but the effect was marginal compared with that of anti-CD4bs antibodies, 42F9 and 49G2 (Fig. 4). The slight increase in the binding affinity of anti-V3 antibodies in I37K mutants may contribute to the enhanced neutralization of C34-resistant mutants. Anti-MPER antibodies were omitted from this analysis because of the MPER epitope inaccessibility to these antibodies in un-triggered envelope [58].

These results indicate that the I37K mutation induces a conformational change in the envelope trimer, which results in the enhancement of antibody binding owing to improved accessibility to epitopes on gp120. The I37K mutation had a drastic effect on the binding affinity of the anti-CD4bs antibodies 42F9 and 49G2, and this may be correlated with the enhanced neutralization of C34-resistant viruses by these antibodies. However, mechanisms other than an increase in the binding affinity are likely responsible for the enhanced neutralization by VRC01 and anti-V3 antibodies.

### Influence of mutations in gp41 on the infectivity and envelope content of mutant virions

It has been suggested that the amount of envelope content per HIV-1 virion may affect the viral infectivity and neutralization sensitivity [38, 59, 60]. It was reported that a 10- to 14-fold rise in the envelope content in an SIV model may lead to a 20- to 500-fold increase in infectivity, which results in resistance to neutralization [59, 61, 62]. Therefore, we investigated the effect of C34-resistance-associated mutations on the viral infectivity and envelope content on the virion surfaces.

**Fig. 5:**
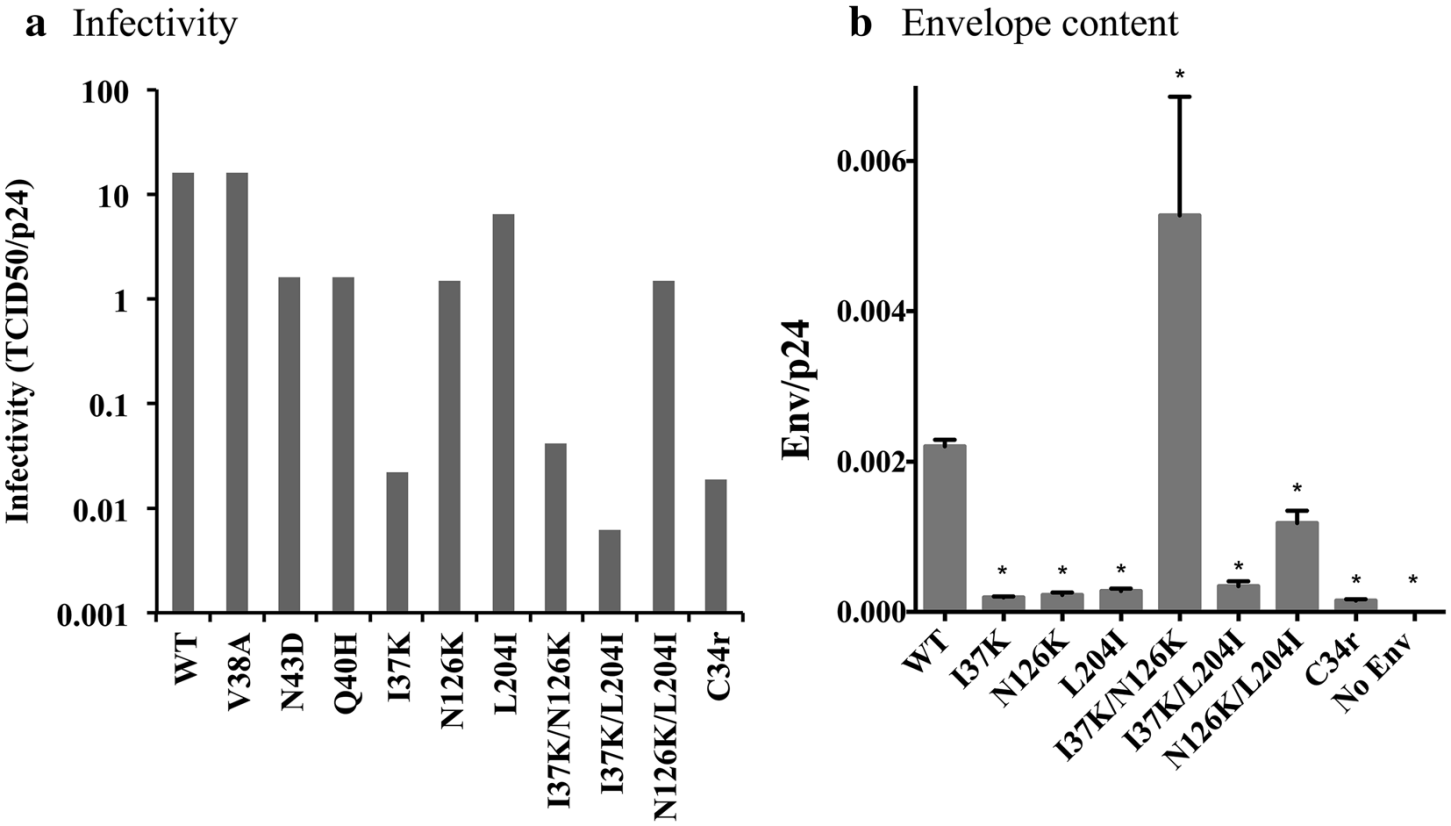
Comparison of infectivity and envelope content among fusion inhibitor-resistant mutants. **a** The infectivity levels of ENF-resistant mutants, V38A, N43D, and Q40H, and of C34-resistant mutants, I37K, N126K, L204I, I37K/N126K, I37K/L204I, N126K/L204I, and C34r, are shown. The TCID_50_ and p24 concentrations were determined by single round infection assays using TZM-bl cells and ELISAs, respectively. Infectivity is shown as the TCID_50_/p24 amount ratio. **b** The envelope content per virion was compared among C34 resistance mutants. The concentrations of envelope and p24 in pseudovirus stocks were determined by ELISAs. As negative control pseudovirus expressing no envelope was used. The envelope content is shown as the envelope/p24 amount ratio. The results are expressed as the means ± SEs of three independent experiments. *Asterisks* correspond to values that are statistically different from those of the WT (*p* < 0.05 as calculated using the Mann–Whitney *U* test)

As shown in Fig. 5a, the infectivity of the C34r mutant was 1000-fold lower than of the WT, which can be compared with the mild effect on infectivity of mutations conferring ENF resistance. The I37K mutant, as well as combination mutants that included I37K, also showed a significant decrease in infectivity compared with the WT, similar to the C34r mutant, suggesting that I37K is the major mutation responsible for the reduced infectivity. Two ENF-resistant mutants, N43D and Q40H, showed low infectivity compared with WT (approximately tenfold), but the infectivity of the V38A mutant was at the same level as the WT. These data demonstrate that I37K is critical for C34 resistance in JR-FL, but its presence imposes a significant fitness cost (Fig. 5a). The effect of N126K on C34 resistance and its fitness cost were lower than that of I37K. We are unable to comment on the role of L204I in fitness because it was evaluated by single round infection assay in this study (14).

We observed a 7- to 11-fold decrease in the envelope content of I37K, N126K, and L204I mutants as compared with WT (Fig. 5b). C34r also showed a 14-fold reduction in the amount of envelope per virion. There was not a consistent pattern in the Env content for combination of these three mutations; I37K/N126K had an over twofold increase in envelope per p24, whereas N126K/L204I showed an approximately twofold decrease. The envelope concentration for I37K/L204I was similar to the envelope content of L204I (6.5-fold decreases). This change in the envelope content on the surfaces of I37K and C34r pseudoviruses may be partially responsible for their reduced infectivity and higher sensitivity to neutralization. The amount of envelope on virion surfaces and its impact on neutralization sensitivity still awaits clarification [38, 59, 60]. Additionally, another limitation of this data is that they measure the monomeric gp120 from lysed viruses, but the number of functional trimers may influence the neutralization sensitivity.

### Slow fusion kinetics of C34r and I37K mutants

**Fig. 6:**
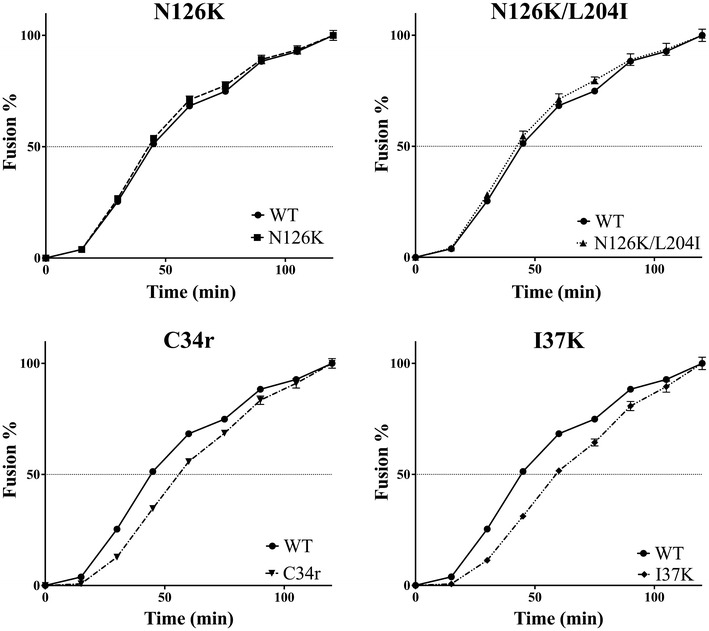
Fusion kinetics of C34-resistant mutants. The effects of N126K, N126K/L204I, C34r, and I37K mutations on fusion kinetics were determined by dual split protein (DSP)-dependent cell–cell fusion assays. *Renilla* luciferase activity, which was detected in fused cells, was monitored periodically, and fusion is expressed as the percentage of fusion after 120 min. The results are shown as the means ± SEs of at least five replicas

It has been suggested that enhancement of neutralization by anti-MPER antibodies is due to slower fusion kinetics, which lead to prolonged exposure of the epitope [25, 63–65]. To test this hypothesis, we performed fusion assays for I37K, N126K, N126K/L204I, and C34r mutants. As shown in Fig. 6, both I37K and C34r have reduced fusion kinetics with respect to WT. However, no such change was apparent for N126K or N126K/L204I mutants (Fig. 6). These data are consistent with the enhanced neutralization sensitivity of C34r and I37K mutants to 10E8 (Fig. 3b).

### Large fluctuations in interfaces between gp41 and gp120 in Env trimer with I37K mutation

**Fig. 7:**
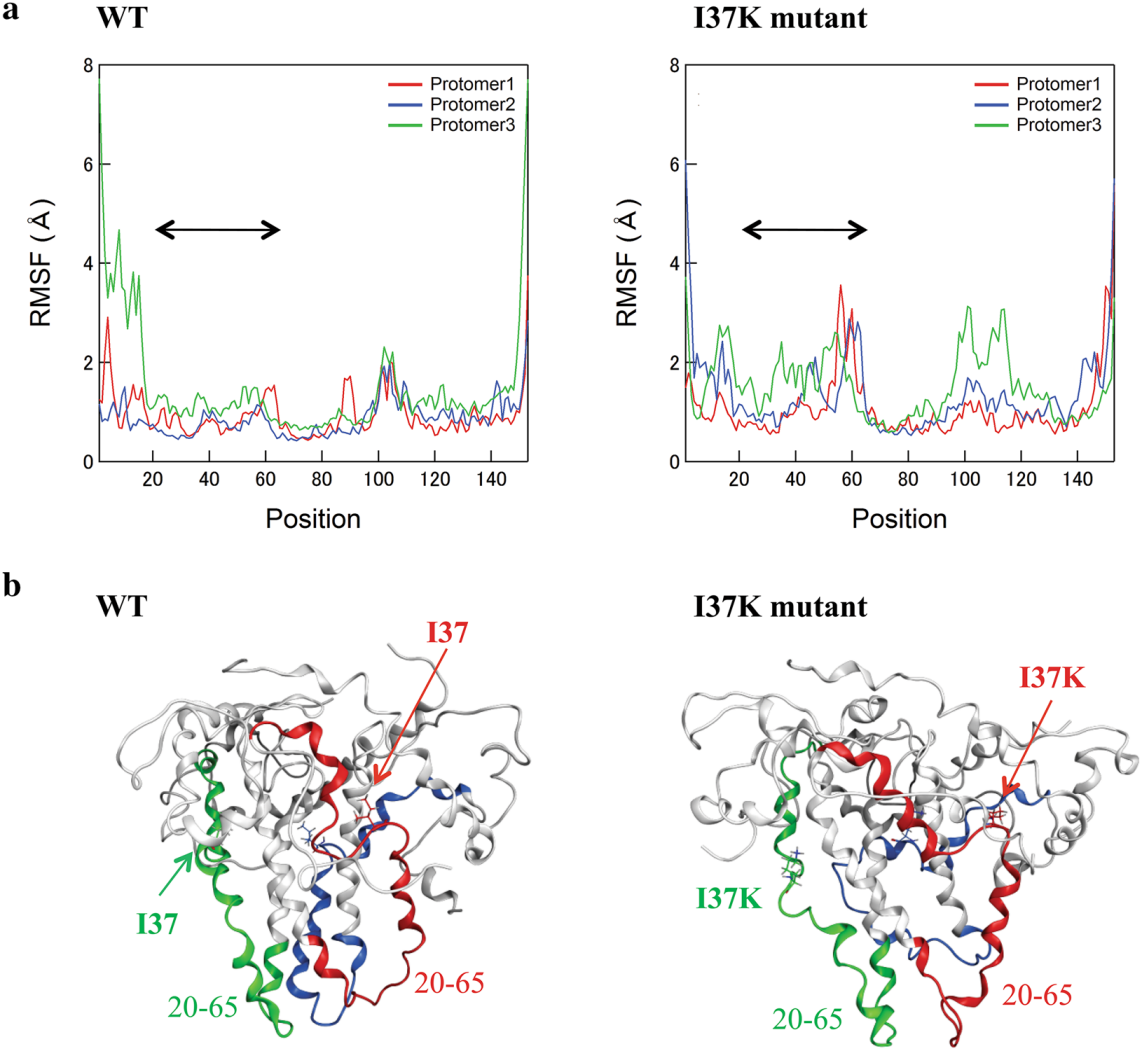
MD simulation of the HIV-1 gp41 trimer. Molecular modeling and MD simulation of the extracellular portion of the HIV-1 JR-FL gp41 trimer with and without a I37K mutation were performed using modules in the Molecular Operating Environment and the AMBER 11 program package [46]. **a** The distribution of the RMSFs of three protomers consisting of the gp41 trimer. *Double-arrow heads* indicate regions between positions 20 and 65 in protomers 1, 2, and 3. **b** Structures of the extracellular portion of the HIV-1 JR-FL gp41 trimer with and without a I37K mutation. The structures at 100 ns of MD simulations are shown. The amino acid residues between positions 20 and 65 in protomers 1 (*red*), 2 (*blue*), and 3 (*green*) are *highlighted*

Our data suggest that the I37K mutation in gp41 has global effects on the physical properties of the gp120/gp41 trimer. The amino acid residue at position 37 of gp41 is located at the interface between gp41 and gp120 in the Env trimer [66]. To gain structural insights into the biological effects of the I37K mutation, we conducted structural modeling and MD simulations of the extracellular portion of HIV-1_JR-FL_ gp41 trimer with and without an I37K substitution. Using 2000 snapshots of the structures from the last 20 ns of each MD simulation, we examined the atomic fluctuations of individual amino acid residues by calculating the RMSF of the Cα atoms [46]. The results revealed a marked increase in the RMSFs of the gp41 with I37K mutations (Fig. 7a). Notably, RMSFs at positions 20–65 in the N-terminal portions of the gp41 extensively increased in all three protomers consisting of the gp41 trimer with the I37K mutation (Fig. 7a, double-headed arrows). Notably, this region is located in the protein–protein interface between gp41 and gp120 in the Env trimer (Fig. 7b) [40]. The results suggest that the I37K mutation induces augmentation of structural fluctuations prominently in the interfaces between gp41 and gp120 in the Env trimer.

### Polyclonal IgG from HIV-1 infected patients can neutralize C34r better than WT JR-FL

To clarify the effect of C34-resistant mutations for the neutralization sensitivity against plasma IgG from HIV-1 infected patients, we examined the neutralization sensitivities of the HIV-1 tier 1 virus BaL, the tier 2 virus JR-FL (WT), and the C34r mutant. Among 11 samples, IgG from ten samples (91 %) neutralized BaL, IgG from four samples (36 %) neutralized WT, and IgG from eight samples (73 %) neutralized the C34r mutant (Table 1). The higher frequency of neutralization for C34r suggests that the contribution of non-neutralizing antibodies, such as antibodies against the CD4bs, became neutralizing against the JR-FL variant containing the C34r mutation (Table 1). The high rate of neutralization by patient IgG samples indicates that the C34r mutant is sensitive to antibodies circulating in HIV-infected patients in vivo.

**Table 1:** Neutralization sensitivity (IC_50_) of BaL (tier 1), JR-FL (tier 2), and C34r mutants by purified IgG from HIV-1 infected patients

Patient ID	IC_50_ (µg/mL)		
	BaL	JR-FL	C34r
Patient 1	27	>300	>300
Patient 2	119	>300	74
Patient 3	114	>300	190
Patient 4	46	240	61
Patient 5	124	>300	>300
Patient 6	>300	>300	>300
Patient 7	178	>300	8.4
Patient 8	11	30	48
Patient 9	6	66	46
Patient 10	18	>300	161
KTS376	9	45	70
Total neutralized	91 %	36 %	73 %

IC_50_ of purified IgGs from HIV-1 infected patients against pseudoviruses with envelope from BaL, JR-FL (WT) and C34-resistant mutants were measured using TZM-bl cells

## Discussion

Here, we report the enhanced neutralization sensitivity to anti-CD4bs, anti-V3, and anti-MPER antibodies of HIV-1 variants that are resistant to the fusion inhibitors C34, SC34, and SC34EK. We have identified I37K in the HR1 region of gp41 as a key mutation for C34 resistance, for the exposure of gp120 epitopes, and for slowing down the fusion process, resulting in enhanced neutralization sensitivity of variants with this mutation. The results of a molecular dynamic simulation support the effect of I37K mutation on enhanced neutralization sensitivity, which is indicated by a fluctuation of the gp41 trimer structure.

Early investigation of the MAb sensitivity of ENF-resistant mutants was performed using YU-2 with ENF-resistance-conferring mutations and antibodies 2G12 (anti-glycan), 17b, 48D (anti-CD4i), b12 (anti-CD4bs), 4E10, and 2F5 (anti-MPER) [25]. They found that anti-MPER antibodies enhanced the neutralization sensitivity of YU-2 containing the ENF-resistance-conferring mutations G36D or V38 M. Here, we employed several broad and conventional antibodies targeting different epitopes against fusion inhibitor-resistant mutants in a JR-FL background and found that mutations in gp41 conferring fusion inhibitor resistance affected the sensitivity of the viruses to antibodies against epitopes on gp120. The bnAbs, namely VRC01 and 10E8, were more potent against fusion inhibitor-resistant mutants. Notably, the tier 2 virus JR-FL mutants containing a single mutation of I37K or N126K in gp41 could both be neutralized by antibodies 42F9 and 49G2, which target CD4bs, even though these antibodies were thought to only be capable of neutralizing tier 1 strains [26]. The emergence of neutralization sensitivity to these conventional antibodies suggests that fusion inhibitor-resistant variants are easily neutralized by the antibodies frequently induced in patients, as evidenced by the neutralization of C34-resistant variants with IgG samples from patients infected with HIV. The drug-resistant mutants that evolve against fusion inhibitor stress usually confer resistance independent of the envelope context and can be used as signature sequences [6]. Thus, it is likely that the humoral immune system of HIV-1-infected patients receiving a fusion inhibitor can counter the emerging fusion inhibitor-resistant mutants. A previous study reported that a point mutation in gp120 makes tier 2 viruses that are similarly sensitive to antibodies as tier 1 viruses [67]. Our data indicate that the mutations in gp41 can make tier 2 viruses sensitive to otherwise non-neutralizing antibodies.

HIV-1-infected individuals possess antibodies against the V3-loop epitope of envelope protein [68]. We found that C34-, SC34-, and SC34EK-resistant mutants were each more sensitive to all the anti-V3 antibodies than the WT virus. This indicates that mutations in gp41 affect the antigenic properties of the V3- loop. A previous study reported that mutations in the gp41 fusion peptide region confer resistance to CCR5 antagonists, which inhibits the interaction of the V3 loop with CCR5 [69]. Conversely, mutations in the V3 loop influence the fusion process and fusion inhibitor binding [63]. Our findings, together with these previous observations, indicate that there is a specific functional interaction between the amino acids of the V3 loop and gp41 [63, 69]. Further study of gp41 mutations in the modulation of co-receptor binding site will shed light on the molecular interplay between co-receptor binding and progress towards fusion.

Our neutralization assay data show that I37K is the key mutation for the observed enhancement in neutralization sensitivity. Furthermore, compared with the WT, the N126K mutant has improved neutralization sensitivity to CD4bs MAbs. A previous study suggested that I37K in the HR1 interferes with the binding of C34 and that N126K in the HR2 enhances the intra-gp41 binding of HR1 and HR2 compared with C34 [14]. Our results suggest that these two mechanisms of C34 resistance also affect the neutralization sensitivity of the virus. However, pseudoviruses containing both I37K and N126K mutations showed neutralization sensitivities that are similar to those of WT. This indicates that the I37K and N126K mutations antagonize the effects on neutralization sensitivity of each other. The effect of I37K, which exposes epitopes for neutralization, may be countered by N126K, which stabilizes the gp41 structure by enhancing the binding of HR1 and HR2. Further structural analysis is required to clarify this antagonistic mechanism.

The L204I mutation is one of the three mutations in C34r, and this mutation is commonly observed in variants that are resistant to C34 derivatives (Additional file 1: Fig. S1) [14, 15]. It makes up the tyrosine-dependent sorting signal in the gp41 cytoplasmic domain and enhances viral replication [14]. Of the antibodies we tested, a single L204I substitution appeared to only affect the neutralization sensitivity to the anti-V3 antibody 16G6. The L204I mutation significantly contributed to neutralization sensitivity when it was combined with other C34-resistance-associated mutations, I37K and N126K. As mentioned earlier, I37K and N126K mutations enhanced the neutralization sensitivity compared with that of WT; however, this effect was antagonized by the combination of I37K and N126K mutations. Hence, in addition to I37K and N126K, L204I likely also has functional importance.

The results of flow cytometry analyses of antibody binding indicate that I37K and N126K mutations have different impacts on untriggered cell surface envelope proteins. Anti-CD4bs antibodies 42F9 and 49G2 bound to the I37K mutant better than they did to the N126K mutant, even though the neutralization sensitivities of these mutants to anti-CD4bs antibodies were similar. Additionally, the sensitivity to sCD4 was higher for the I37K mutant than it was for the N126K mutant. These results suggest that the I37K mutation may change the Env structure to an open conformation, in which antibodies can access the epitopes that are hidden inside in the closed conformation [70]. The findings from our MD simulation of the gp41 trimer support this hypothesis. Our study reveals that the I37K mutation can increase the structural fluctuations of gp41 protomers, prominently those in the regions corresponding to the gp41–gp120 interfaces in Env trimer. Because structural fluctuations of the protein play key roles in its molecular interactions [71–73], it is possible that the increases in the fluctuations of the gp41–gp120 interfaces cause an attenuation of the structural compaction and stability of the gp120/gp41 trimer. Such changes in turn would open otherwise sterically hindered neutralization epitopes of gp120 and gp41. Unfortunately, the mechanism by which the N126K mutation enhances neutralization sensitivity is unclear. It is possible that N126K may reduce other structural interference to epitope access on the virion surface during the viral entry step. The lack of relationship between the neutralization sensitivity and the envelope–antibody binding may be owing to antigenic differences between the envelope expressed on the cell surface and the virion surface, as observed in previous investigations [37, 74].

Another effect of gp41 mutations was reduced viral infectivity and envelope content especially for mutants bearing I37K mutation. Infectivity and envelope content can be affected by enhanced gp120 shedding due to destabilization of the interaction of gp120 and gp41 [75]. However, envelope content on virion surface did not correlate with envelope expression level on cell surface (Additional file 3: Fig. S3). Moreover, our preliminary analysis of spontaneous gp120 shedding indicated that most of gp120 was maintained on the surface of cells expressing WT and mutant envelopes, but was not released to supernatant (Additional file 4: Fig. S4). Further research is required to clarify the mechanism of reduced viral infectivity and envelope content.

Unlike ENF resistant mutations C34-, SC34- and SC34EK-resistant mutations have not been studied in clinical setup, marking a major limitation of current study. The mutations obtained by in vitro passages, especially I37K, which has significantly decreased infectivity, may be disadvantageous to viral replication in vivo. However, occurrence of N126K mutation has been observed in ENF-treated patients [53], suggesting that these mutations conferring resistance to C34 derivative fusion inhibitors may arise in HIV-1-infected patients.

## Conclusions

HIV-1 envelope is known for its hypervariability, glycan shield, conformational dynamics, and covered conserved regions, which make it one of the most complex proteins known to date [66, 76]. These complex conformational dynamics make it difficult to understand the functions of the HIV-1 envelope based solely on rigid structure or biochemical analyses [77]. Thus, mutational analyses, especially those developed against drug responses, have and will provide important insights into the dynamics of envelope glycoprotein [67, 78]. In summary, here, we report the enhancement of neutralization sensitivity in the next-generation fusion inhibitor-resistant mutants to neutralizing antibodies. We observed that, along with bnAbs, conventional patient-derived IgGs could neutralize the tested fusion inhibitor-resistant mutants. We have also identified the impact of the I37K and N126K mutations on epitopes in both gp120 and gp41. The results from our attempt to understand the mechanism responsible for the observed neutralization sensitivity changes indicate that the conformational unmasking of envelope glycoprotein affects the neutralization sensitivity in a very complex manner. Recent advances in the exploration of bnAbs indicate that, in the near future, HIV-1 researchers will be looking for drugs to administer in combination with neutralizing antibodies. In past decades, several fusion inhibitors have been designed based on different parameters, but very few have been tested in human subjects. We suggest that next-generation fusion inhibitors in combination with antibodies should be considered as the next-generation combined anti-retroviral therapy regimen.

## Additional files

10.1186/s12977-016-0304-7 Alignment of HIV-1 envelope the gp41 amino acids sequences investigated in this study.
10.1186/s12977-016-0304-7 Sensitivity of fusion inhibitor-resistant JR-FL variants against their corresponding fusion inhibitors. Single round infection assays were used to determine the sensitivity of fusion inhibitor-resistant JR-FL variants against their corresponding fusion inhibitors. Three mutants, V38A, Q40H, and N43D, were examined for sensitivity to ENF (upper left graph). Sensitivity to C34 was analyzed using C34r (I37K/N126K/L204I) and the related double and single mutants, N126K/L204I, N126K, and I37K (upper right graph). Mutants, SC34r and SC34EKr, were examined for sensitivity to SC34 and SC34EK, respectively (lower graphs). In each graph, the x-axis represents the log concentration of the fusion inhibitor in nM and the y-axis represents the percent inhibition compared with the corresponding no inhibitor control. The results are shown as the means ± standard errors of three independent experiments.
10.1186/s12977-016-0304-7 Reactivity of antibodies, VRC01, 42F9, 49G2, b12, KD247, 16G6, 0.5γ and 2G12 to WT and mutant envelopes. Reactivity of antibodies to envelopes was analyzed using flow cytometer, and shown as MFI. Cells expressing Env from SIVsmE543-3 was used as a negative control (SIV).
10.1186/s12977-016-0304-7 Spontaneous gp120 shedding from cell surface. The susceptibility of gp41 mutants to spontaneously shed gp120 was determined by flow cytometry and ELISA as described previously [79]. Briefly, culture medium of transiently transfected envelope expressing cells was exchanged for fresh medium containing Brefeldin A (BioLegend) and 0.2 % Sodium azide. Cells were then incubated for 15 h at 37˚C, 5 % CO_2_. (**a**) Level of envelope expression before and after incubation was compared by staining with 2G12. (**b**) Amount of gp120 released during incubation was determined by gp120 capture ELISA. As a positive control, cells expressing WT envelope was incubated with 20 µg/ml sCD4, which trigger gp120 shedding. Cells expressing SIV Env (SIV) and no Env (No Env) were used as negative control. The results are shown as the means ± standard errors of four replicas.
